# Functional and Structural Consequences of Damaging Single Nucleotide Polymorphisms in Human Prostate Cancer Predisposition Gene RNASEL

**DOI:** 10.1155/2015/271458

**Published:** 2015-07-08

**Authors:** Amit Datta, Md. Habibul Hasan Mazumder, Afrin Sultana Chowdhury, Md. Anayet Hasan

**Affiliations:** Department of Genetic Engineering and Biotechnology, Faculty of Biological Sciences, University of Chittagong, Chittagong 4331, Bangladesh

## Abstract

A commonly diagnosed cancer, prostate cancer (PrCa), is being regulated by the gene RNASEL previously known as PRCA1 codes for ribonuclease L which is an integral part of interferon regulated system that mediates antiviral and antiproliferative role of the interferons. Both somatic and germline mutations have been implicated to cause prostate cancer. With an array of available Single Nucleotide Polymorphism data on dbSNP this study is designed to sort out functional SNPs in RNASEL by implementing different authentic computational tools such as SIFT, PolyPhen, SNPs&GO, Fathmm, ConSurf, UTRScan, PDBsum, Tm-Align, I-Mutant, and Project HOPE for functional and structural assessment, solvent accessibility, molecular dynamics, and energy minimization study. Among 794 RNASEL SNP entries 124 SNPs were found nonsynonymous from which SIFT predicted 13 nsSNPs as nontolerable whereas PolyPhen-2 predicted 28. SNPs found on the 3′ and 5′ UTR were also assessed. By analyzing six tools having different perspectives an aggregate result was produced where nine nsSNPs were found to be most likely to exert deleterious effect. 3D models of mutated proteins were generated to determine the functional and structural effect of the mutations on ribonuclease L. The initial findings were reinforced by the results from I-Mutant and Project HOPE as these tools predicted significant structural and functional instability of the mutated proteins. Expasy-ProSit tool defined the mutations to be situated in the functional domains of the protein. Considering previous analysis this study revealed a conclusive result deducing the available SNP data on the database by identifying the most damaging three nsSNP rs151296858 (G59S), rs145415894 (A276V), and rs35896902 (R592H). As such studies involving polymorphisms of RNASEL were none to be found, the results of the current study would certainly be helpful in future prospects concerning prostate cancer in males.

## 1. Introduction

Single Nucleotide Polymorphism, also known as SNP, accounts for the most common form of genetic mutation in human. It has been reported that ~93% of all human genes represent at least one SNP [1]. Therefore, they are liable for generating the majority of biological variations among individuals. An understanding of the relationship between these genetic variations and their phenotypic effects could therefore be a step toward exploring the causes of various disorders or diseases. SNPs can fall within the coding regions (coding SNPs) or noncoding regions of genes (noncoding SNPs), or in the intergenic region between two genes [2, 3]. While the two others are quite natural in the human genome and phenotypically neutral [1, 4], nonsynonymous coding SNPs (nsSNPs) are thought to have the principal impact on phenotype by changing the protein sequence. As they cause amino acid alteration in the corresponding protein product, it may exert deleterious effects on the structure, function, solubility, or stability of proteins [5, 6]. Beside these the nsSNPs perturb gene regulation by modifying DNA and transcriptional binding factors [6–9] and the maintenance of the formational integrity of cells and tissues [10]. Thus it is likely that nsSNPs play a major role in the functional diversity coded proteins in human populations and often associated with human diseases. Indeed, earlier studies have revealed that more than 50% of the mutations associated with inherited genetic disorders are resulted by nsSNPs [11–13]. Recently, many researchers have focused on nsSNPs in cancer causing genes. The recent studies have identified multiple nsSNPs that influence susceptibility to infection, as well as the development of inflammatory disorders and autoimmune diseases [4–9]. Nonetheless, because innate immune genes are often highly polymorphic, many nsSNPs in these genes remain uncharacterized.

Prostate cancer (PRCA) is one of the most commonly diagnosed cancers worldwide, mostly in developed countries [14]. In the United States it is the second leading cause of cancer death in males [15]. Currently, no permitted curative therapies are available for prostate cancer that has been metastasized. In the United States it is the second leading cause of cancer death in males [15]. Therefore, researches have focused to detect newer suitable solution for controlling prostate cancer and generating new potential targets for therapy. A fraction of PRCA patients belong to the hereditary prostate cancer (HPC) families. Linkage analyses in HPC families have predicted that PRCA susceptibility genes are harbored in multiple genetic loci, including* HPC1*, at 1q24-q25;* ELAC2/HPC2*, at 17p11;* PCAP*, at 1q42.2-q43;* HPCX*, at Xq27-q28;* CAPB*, at 1p36; and* HPC20*, at 20q13 [16]. As study shows that genetic background has remarkable contribution to cause prostate cancer [15], both the identification and treatment of cancer would potentially aid from the detection of new genes that are particularly expressed in prostate cancer. Among the large number of loci reported, the* HPC1* locus, at 1q24-q25, harbors the gene RNASEL (encoding ribonuclease L or RNase L) a recently proposed candidate for the hereditary prostate cancer (HPC) gene [17].

RNase L is a ubiquitously expressed, 2′, 5′-oligoadenylate (2-5A) dependent endoribonuclease that involves the antiviral action and proapoptosis activity in interferon [18, 19]. In virus infected and INF treated cells, RNase L probably shows its antiviral effects through a combination of activities which include direct cleavage of the single-stranded viral RNAs, suppression of protein synthesis via the disruption of rRNA, beginning of apoptosis, and induction of associated antiviral genes. RNaseL induced apoptosis is the ultimate result of a JNK-dependent stress-response pathway which leads to the release of cytochrome c from mitochondria and caspase-dependent cell death (apoptosis). Therefore, RNase L activation could lead to the elimination of virus infected cells under different circumstances. It might play a central role also in the regulation of mRNA turnover. In control of viral and cellular growth, the role of the 2-5A system suggests that defects in the 2-5A-dependent RNASEL gene could result in decreased immunity to virus infections and cancer [20]. Again, RNase L is involved in the regulation of cell proliferation through the interferon-regulated 2-5A pathway and therefore had been suggested as a tumor suppressor gene. In that connection, a study found loss of heterozygosity and loss of RNase L protein in the microdissected tumors with a germline mutation [21]. This indicates that RNASEL activity was reduced in lymphoblasts from heterozygous individuals compared with family members who were homozygous with respect to the wild type allele. Thus, germline mutations in RNASEL might have diagnostic value, and the 2-5A pathway may present opportunities for developing therapies for the prostate cancer patients. In that regard, recent studies on hereditary prostate cancer (HPC1), positioning at the locus 1q24-q25, found two rare heterozygous inactivating germline mutations in RNASEL linked to HPC1.

From the last few years, computational approaches have been widely used to detect the impact of deleterious nsSNPs in candidate genes by analyzing data such as conservation of sequences across species [22], physicochemical properties of the polypeptides [23, 24], and structural attributes [25].

By following computational algorithms, the functional SNPs out of a vast range of disease susceptible SNPs of ATM gene [26],* BRCA1 *gene, and* BRAF *gene [27] have successfully divided based on their structural and functional properties. In recent years computational approaches have been adopted by many researchers in cancer studies either as a part of large population data analysis or only for predicting most deleterious SNPs from the large datasets. Similar work has been done extensively for breast cancer associated genes BRCA1 [28] and BRCA2 [29] where more than thousand SNPs have been analyzed altogether. Insulin-like growth factor 1 receptor (IGF1R) is another gene associated with both breast cancer and prostate cancer and polymorphisms in the IGF1R receptor are found [30] which cause instability of the receptor protein. Moreover, SNPs which can increase disease predisposition in colorectal cancer (HPNCC gene and MCAK gene) [31, 32], haemoglobinopathies (beta-globin gene) [33], and breast cancer (BARD1 gene) [34] have been predicted in computational studies. Several oncogenes such as cyclin-dependent kinase 7 (CDK7) gene [35], ErbB3 gene [36], polo-like kinase 1 (PLK1) [37], centromere-associated protein-E (CENP-E) [38], and centrosomal protein of 63 kDa (CEP63) [39] involved in cell cycle regulation and cell division are reported to contain damaging SNPs in corresponding* in silico *studies.

Basing on the convincing data which indicates the wide involvement of RNASEL gene in varied range of human diseases, its functional genomics depend on mutation analysis which is conceived to provide key advances in disease diagnosis and therapy. But, the* in silico* analysis of noncoding SNPs (intronic, exonic and 5′ and 3′ UTR SNPs) and coding SNPs (nsSNPs and sSNPs in exonic regions) in our candidate HPC1 gene remains unpredicted to date.

Hence, in the current investigation the* in silico *analysis of RNASEL gene has been carried out in order to characterize the deleterious mutations. Our investigational study involved (i) retrieval of SNPs in* RNASEL *gene from available databases, (ii) allocating the deleterious nsSNPs to their phenotypic effects, based on sequence and structure-based homology search and identifying the regulatory nsSNPs that can alter the splicing and gene expression patterns, (iii) predicting the specific effects of the substitutions of amino acids on secondary structures by means of solvent accessibility and stability, (iv) and prediction of change in the domain structures due to the mutations. The above* in silico *approaches offer a total of 9 high-risk nsSNPs sorted out from the NCBI SNP database. This study is the first extensive* in silico* analysis of the RNASEL gene and will establish a strong foundation for structure-function and population-based studies in future.

## 2. Materials and Methods

**Figure 1:**
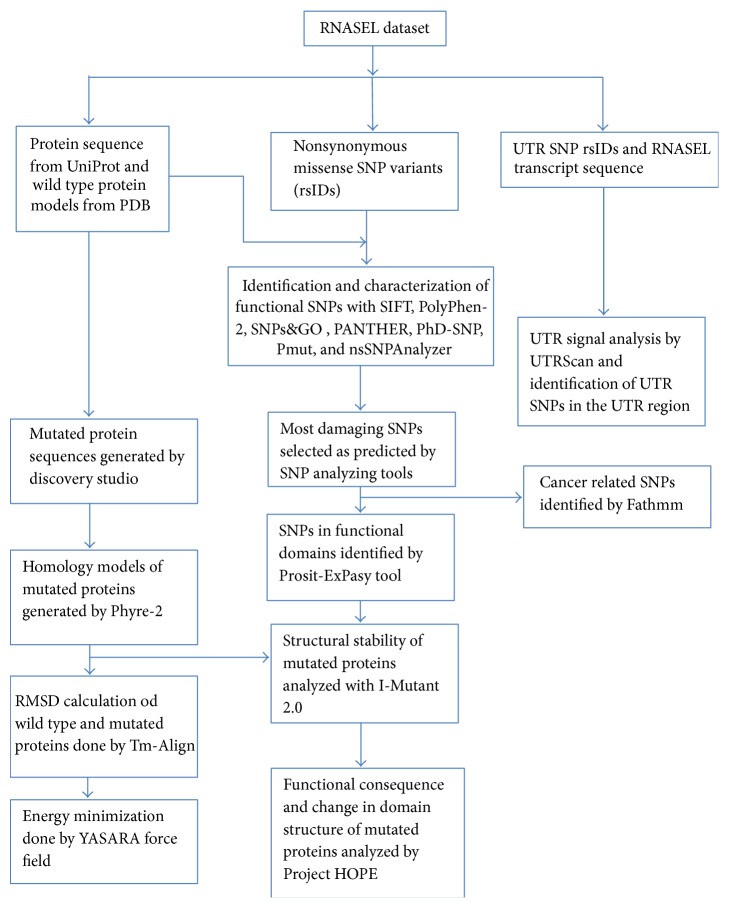
The Flow Chart depicting the overall process of identification and characterization of damaging SNPs in RNASEL along with the structural and functional consequence analysis upon mutation.

The Flow Chart depicts the overall process of identification and characterization of damaging SNPs in RNASEL along with the structural and functional consequence analysis upon mutation (Figure 1).

### 2.1. Retrieval of SNP Datasets

The data on human RNASEL gene was derived from web-based data sources such as Online Mendelian Inheritance in Man (OMIM) [40], the SNPs information (protein accession number and SNP ID) of the RNASEL gene was retrieved from the NCBI dbSNP (Database of Single Nucleotide Polymorphism) [41], and the protein sequence and protein structure were retrieved from Uniprot (Universal Protein Resource) [42] and RCSB protein databank [43] subsequently.

### 2.2. Analysis of Functional Consequences of nsSNPs

Sorting Intolerant from Tolerant (SIFT) [44] is a tool that can detect the deleterious coding nonsynonymous SNPs. This program presumes that major amino acids will be conserved in the protein family and changes at specific positions tend to be predicted as deleterious [28, 44]. During mutagenesis studies in human SIFT can differentiate functionally neutral and deleterious polymorphisms [45]. The algorithms for the SIFT program use SWISSPROT, nr, and TrEMBL databases to find homologous sequences. The threshold for the intolerance index is ≥0.05. In this study, the identification numbers (rsIDs) of each SNP of human RNASEL gene obtained from NCBI were submitted as a query sequence to SIFT for homology searching. The SIFT value ≤0.05 indicates the deleterious effect of nonsynonymous variants on protein function.

### 2.3. Prediction of Functional Consequences of Coding nsSNPs by Structural Homology-Based Method

To understand the functional significance of a protein it is crucial to analyze the damaged coding nonsynonymous SNPs at the structural level [46, 47]. Polymorphism Phenotyping-2 or PolyPhen-2 [11] which is a probabilistic classifier that computes functional impact of an allele change by Naive Bayes, a set of supervised learning algorithms, was used to determine structural consequences. Query was submitted in the form of protein sequence along with mutational position and two amino acid variants. PolyPhen categorizes the SNPs as benign, possibly damaging, or probably damaging on the basis of site-specific sequence conservation by estimating the position-specific independent count (PSIC) score for every variant and also calculates the score difference between variants [11]. The higher the PSIC score difference is, the higher the functional impact a specific amino acid substitution is likely to have.

### 2.4. Characterization of Functional nsSNPs

For characterization of functional nsSNPs SNP&GO was used. It accumulates five functional tools SNP&GO [48], PANTHER [49], PHD-SNP [50], nsSNP Analyzer [51], and P-MUT [52]. Single Nucleotide Polymorphism Database (SNPs) and Gene Ontology (GO) and Predictor of Human Deleterious Single Nucleotide Polymorphisms (PhD-SNP) use support vector machine (SVM) based analyzing method, where the Protein ANalysis THrough Evolutionary Relationships (PANTHER) estimates the function of coding nsSNPs by calculating the subPSEC (substitution position-specific evolutionary conservation) score [48, 53]. nsSNPAnalyzer uses information contained in the multiple sequence alignment and information contained in the 3D structure to make predictions. Lastly, Pmut is based on the use of different kinds of sequence information to label mutations and neural networks to process this information. FASTA sequence was inputted and result was based on the differences among disease related and neutral variations of protein sequence. Probability score higher than 0.5 reveals the disease related effect of mutation on the protein function [18].

### 2.5. Prediction of Cancer Promoting Mutations

The cancer-associated variants were predicted by using the Functional Analysis through Hidden Markov Models (Fathmm) which combine sequence conservation within hidden Markov models (HMMs) [54]. Fathmm server is a high-throughput web server that can predict phenotypic, molecular, and functional consequences of protein variants both on coding and noncoding variants. It uses two algorithms unweighted, sequence/conservation based and weighted, combined by sequence conservation with pathogenicity weights. For Fathmm server the default prediction threshold is −0.75 where prediction with score less than this indicates that the mutation is potentially associated with cancer. Cancer promoting mutations plays critical role in cell regulation and mutations falling in the conserved region can downweight the nature of the domain.

### 2.6. Identification of Functional SNPs in Conserved Regions

Evolutionary conservation of amino acid substitution was predicted by ConSurf web server [55] by using a Bayesian algorithm (conservation scores: 1–4 variable, 5-6 intermediate, and 7–9 conserved) [56, 57]. Protein structure of RNase L was submitted and the conserved regions were predicted by means of colouring scheme and conservation score. Functional and structural residues were also predicted. Highly conserved amino acids located at high-risk nsSNP sites were nominated for further analysis.

### 2.7. Scanning of UTR SNPs

The 5′ and 3′ untranslated regions (UTRs) have important roles in the posttranscriptional regulation of gene expression, translational efficiency, and stability [58]. To predict the functional SNPs we used UTRScan [59], a pattern matcher tool that searches protein or nucleotide sequences to find UTR motifs collected in the UTRsite. UTRsite derives data from UTRdb, a curated database that updates UTR datasets through primary data mining and experimental validation [60, 61]. After performing with probable different nucleotide at an SNP position if different sequences for each UTR SNP are found to have varying functional patterns, this UTR SNP is expected to have functional impact. To perform this primary FASTA format data was submitted and the results showed predicted UTRs at the specific region (5′ and 3′).

### 2.8. Modeling of the Mutated Protein

To find proteins related to RNASEL gene The EMBL-EBI Web-based tool PDBsum [62, 63] was used that performs a FASTA search against sequences submitted in the protein data bank (PDB) to obtain the closest matches. The RNASEL FASTA sequence was given in the query space and from the results closest matches were selected.

Virtual Mutation (VM) refers to the substitution of a single or multiple amino acids in the atomic 3D model of the molecule [64, 65]. Accelrys Discovery Studio 4.0 was used to generate mutated sequence for the corresponding amino acid substitutions [66, 67]. Regenerated mutant sequences were used further for mutant modeling. Determining properties and three-dimensional structure of a macromolecule, such as enzymes, antibodies, DNA, or RNA, is a vital element to a wide range of research activities. The modeling of mutated proteins were performed through, Phyre2 (Protein Homology/Analogy Recognition Engine) [68], the most popular online protein fold identification server. Phyre2 selects the best suited template and creates a protein model through sequential steps, such as profile construction, similarity analysis, and structural properties. Intensive mode of protein modeling was selected to get an accurate model. The input data of the proteins were in FASTA format.

### 2.9. Energy Minimization and RMSD Calculation of the Protein Models

Tm-Align is a sequence-order independent protein structure comparison algorithm. Tm-Align performs optimized residue to residue alignment based on structural similarity using dynamic programming iterations. This server was used for RMSD calculation of the protein structures [69].

YASARA-minimization server was used to perform the energy minimization of the mutated protein models. Yet Another Scientific Artificial Reality Application (YASARA) is a molecular graphics, modeling, and simulation program with diverged application where YASARA-minimization server uses YASARA force field for energy minimization that can optimize the damage of the mutant proteins and thus precisely calculates the energy. To perform this strategy the pdb file of the mutant proteins were inserted as input data and the result was further analyzed for forthcoming steps [70].

### 2.10. Prediction of Change in Stability upon Mutation

I-Mutant 2.0 server was used to predict the change in stability due to mutations. I-Mutant is a support vector machine (SVM) based tool server. This tool can automatically predict the change in structural stability analyzing the structure or the sequence of the protein. I-Mutant 2.0 can be used as a classifier for predicting the sign of protein stability upon mutation and a regression estimator which predicts the change in Gibbs free energy. The resulting DDG value is the difference between the Gibbs free energy of mutated protein and wild type protein in kcal/mol [71].

### 2.11. Prediction of Structural Effects upon Mutation

Project HOPE was utilized to see the structural effect of the amino acid substitutions. Project Have yOur Protein Explained (HOPE) was used for molecular dynamics simulation to observe the effect of the mutations on the structure of RNASEL. This web server, after given input of the protein sequence, performs a BLAST against the PDB and builds a homology model of the protein if possible through YASARA and collects tertiary structure information from What IF web services and then access the UniProt database for sequence features like active site, domains and motifs, and so forth. Finally to predict the features of the protein it uses Distributed Annotation System (DAS) servers which can exchange annotations on genomic and protein sequences [72].

## 3. Results

### 3.1. SNP Dataset

The polymorphism data is available from several databases; NCBI dbSNP database, the Ensembl genome browser, and the UniProt database for such. The NCBI dbSNP database is the most extensive SNP database of the aforementioned databases, but it contains both validated and nonvalidated polymorphisms. Despite this drawback the SNP data for RNASEL gene was collected from dbSNP because it houses the largest polymorphism database [73].

The dbSNP contains total of 794 SNPs for the gene RNASEL. Of the 794 SNPs 122 were missense SNPs and 151 were in the UTRs. Among 151 UTR SNPs 142 SNPs were in the 3′ UTR and 9 SNPs were found in the 5′ UTR. There were 2 nonsense/stop gained SNPs, but only the missense and 3′ and 5′ UTR SNPs were selected for further analysis.

### 3.2. Nonsynonymous SNP Analysis

A sequence homology based tool SIFT can determine the conservation of a particular position of any amino acids in a given protein sequence. SIFT aligns paralogous and orthologous protein sequences to determine the influence of an amino acid substitution considering its functional significance and physical properties. SIFT has been confirmed to be sufficiently accurate to detect disease related SNPs by predicting the known disease related SNPs from the database compromising only a 20% false positive result. Furthermore, a large number of SNP data available in the database lack structural information related to the SNP but SIFT algorithm performs analysis using the sequence data, so it can predict a significantly large number of SNPs from the database. As a result SIFT provides advantages over other deleterious SNP prediction algorithms [44, 74].

SIFT takes rsID of SNPs as input and a  .txt file was uploaded containing the rsIDs to the SIFT server. SIFT calculates the tolerance index (TI) of a particular amino acid substitution. SIFT score is categorized as tolerant (0.201–1.00) or intolerant (0.051–0.10) and borderline (0.101–0.20). So, a Single Nucleotide Polymorphisms functional consequence is inversely proportional to the tolerance index (TI). Among 122 submitted nsSNP rsIDs from dbSNP SIFT analyzed 11 nsSNPs to bear a deleterious effect with TI score ≤0.05, results are shown in Table 1. The corresponding 4 nsSNPs, rs35896902, rs145415894, rs145787003, and rs182539049, had a tolerance index of 0.01. The following 5 rsIDs, rs114166108, rs142939718, rs145581875, rs146781980, and rs190359946, had the tolerance index 0.02 and rs143374873; rs150721457 had a score of 0.03 and 0.05, respectively. All of the 11 amino acid substitutions were mutually exclusive.

### 3.3. Prediction of Functional Modification of Coding nsSNPs

nsSNPs with the potential to cause structural modifications due to the amino acid substitution were determined through PolyPhen program. It can predict the structural fate of any amino acid substitution with approximately 82% accuracy by applying some empirical rules on the sequence of the protein, compromising only an 8% chance of false positive result. The PolyPhen server functions by accessing UniProtKB nonredundant protein sequence and PDB/DSSP protein structure database. PolyPhen uses the TMHMM algorithm for sequence based characterization of the sequence, Coils2 program and SignalP program for transmembrane, coiled coil, and signal peptide regions prediction of the protein sequence. For identifying the homologous proteins, PolyPhen performs a BLAST query of the given protein sequence and calculates position-specific independent count (PSIC) for every input variant and estimates the difference between the variant scores; the score difference of more than 0.339 is detrimental. PolyPhen scores were allocated probably damaging (2.00 or more), possibly damaging (1.40–1.90), potentially damaging (1.20–1.50), benign (0.00–0.90), and borderline (1.00–1.20). To determine the structural effects PolyPhen also does a BLAST query of the input protein sequence against PDB and PQS structure databases and maps the query substitutions to known 3D structures. The solvent accessible surface areas of the mapped amino acids are attained from DSSP database to generate a complete report of the deleterious effect of the nsSNPs on protein structure [11].

A total of 122 nsSNP rsIDs were submitted to the PolyPhen server and in the resulting output 27 amino acid substitutions have been reported to be probably damaging with PSIC score range from 0.539 to 1 as shown in Table 2. Eight nsSNPs (rs114166108, rs182539049, rs146781980, rs145415894, rs142939718, rs145787003, rs35896902, and rs150721457) were identified by SIFT as deleterious, also marked to be damaging by PolyPhen-2 program as well.

To further validate the results of the tools used beforehand we analyzed the nsSNPs with the following* in silico* SNP prediction algorithms: nsSNP analyzer, PhD-SNP, PANTHER, P-MUT, and SNPs&GO. Primarily we selected the nsSNPs which are marked as deleterious by both SIFT and Poly-Phen-2 server. The SIFT and PolyPhen server is distinctly precise so we combined the prediction of the five abovementioned tools and compared them with the result of SIFT and PolyPhen server. In the combined result 6 (rs114166108, rs182539049, rs145415894, rs142939718, rs35896902, and rs150721457) of the previously selected 8 nsSNPs (predicted deleterious by SIFT and PolyPhen) were predicted as disease related by at least 3 out of the 5 tools. Two nsSNPs, rs142939718 and rs35896902, showed positive results in all the 7 tools. Though predicted by SIFT as tolerated we also selected rs151296858, rs138685180, and rs147890567 for further analysis as found deleterious by at least 6 SNP analyzers. So, in the final result the 9 nsSNPs that came out through the process are rs151296858, rs114166108, rs182539049, rs138685180, rs145415894, rs142939718, rs35896902, rs147890567, and rs150721457. The results are shown in Table 3.

### 3.4. Conservation Profile of High-Risk Nonsynonymous SNPs

Functional sites of proteins such as the enzymatic sites and protein-protein interaction sites are important for biological processes. Amino acids located in this biologically active sites tend to be highly conserved, more than other residues. Any substitution of these functionally involved residues generally leads towards the complete loss of biological functions and render severe deleterious effects compared to other polymorphisms of nonconserved site [13, 75].

ConSurf web server was used to calculate the degree of evolutionary conservation at each amino acid position of the RNase L protein. ConSurf identifies putative structural and functional residues and determine their evolutionary conservation by applying empirical Bayesian method [55].

Although a complete analysis was done we focused on the conservation profile of the selected 9 high-risk nsSNP locations. The analysis showed that residues G59, P62, L184 L224, A276, L361, R592, T595, and I 673 are highly conserved having the conservation score between 7 and 9. According to ConSurf the residues A276 and L361 are conserved and buried denoting them as critical structural residues and G59, P62, and T592 are conserved exposed residues emphasizing their critical functional importance. The highly conserved residues are identified as functional or structural based on their location relative to the protein surface or the protein core. To identify the functional and structural sites ConSurf combines evolutionary data and solvent accessibility predictions [76].  Table 4 shows the result from ConSurf.

In the analysis 5 residues (G59, P62, A276, L361, and T592) that were detected as essential functional and structural residues correspond to the nsSNPs which were predicted to be deleterious by at least 6 out of 7 SNP analyzing algorithms. So, corresponding mutations G59S, P62S, A276V, L361P, and T592H to the nsSNPs rs151296858, rs114166108, rs145415894, rs142939718, and rs35896902 can severely disrupt structural and functional properties of RNase L.

### 3.5. Identifying Cancer Associated Variants

From Fathmm, for Q05823 cancer association was predicted for the amino acid substitution P62S, G59S, and A276V with the scores −1.58, −1.82, and −1.53, respectively.

### 3.6. Functional SNPs in UTR Found by the UTRscan Server

Gene expression is affected by the SNPs in the 3′ UTR region, purposely due to defective ribosomal RNA translation or by affecting RNA half-life [77]. The UTRscan server was used to analyze 142 3′UTR SNPs and 9 5′ UTR SNPs of PRCA gene collected from dbSNP database. The UTRscan server looks for patterns in the UTR database for regulatory region motifs and according to the given SNP information predicts if any matched regulatory region is damaged [78]. UTRscan found 5 UTRsite motif matches in the RNASEL transcript. Total 20 matches were found for 5 motifs. The results are shown in Table 5.

### 3.7. Comparative Modeling of High-Risk Nonsynonymous SNPs

As the SAAPdb was unavailable for maintenance we were unable to map the mutations in the protein structure. To find out the structure of the closest related proteins we submitted the structure to the NCBI protein BLAST tool and performed BLAST against the Protein Database (PDB). The BLAST result showed two PDB entries 4OAU and 4OAV with 100% identity. We selected the structure with PDB id 4OAV and manually scanned the structure and with YASARA view mutation tool carried out the mutations (G59S, P62S, L184S, L224P, A276V, L361P, R592H, T595M, and I673T) separately on the RNase L protein. Energy minimization results from YASARA-minimization server showed decreased free energy for all the mutant models than the wild type models. The results shown in Table 6 RMSD calculation was done by Tm-Align tool where the results showed a RMSD score of 2.24 for the mutation R59H and lowest .42 for G59S. Mutation A276V in the mutant model scored 1.94 in the RMSD calculation. These results indicate significant change in the structure of the protein that can hamper its natural function.

### 3.8. Prediction of Protein Structural Stability

We used the neural network based routine tool I-Mutant 2.0 for analyzing the potential alteration in protein stability due to mutations. This tool took input of the mutated protein models derived from PHYRE-2 server in  .pdb format. I-Mutant 2.0 generates results based on ProTherm database which contains the most extensive amount of thermodynamic experimental data on free energy alterations of proteins stability owing to mutations. 80% or 70% accurate prediction can be achieved by using protein structure or sequence, respectively, by this tool. In addition to that this tool also provides the score of free energy change prediction due to mutations incorporating the energy based FOLD-X tool. This increases the precision to 93% on one-third of the database if FOLD-X analysis is incorporated along with I-Mutant [73].

Models with following mutations G59S, P62S, L184S, L224P, A276V, L361P, R592H, T595M, and I673T were submitted to the server for DDG stability prediction and RSA calculation. All the mutations decreased protein stability except A76V, which is shown to be increasing structural stability but with a reliability index score 3. Mutation I673T accounted for the lowest DDG value (−2.46 kcal/mol) followed by L184S (−2.24 kcal/mol). All other mutations' DDG values ranged from −0.54 kcal/mol to −2.13 kcal/mol; this suggests decreased protein stability, due to DDG values being less than 0. The results are shown in Table 7.

### 3.9. Effect of Mutations on Domain Structures of RNSEL and Their Functional Consequences

The Prosit-ExPasy tool was used to search for domain structures in RNase L and map the mutations in the domains for determining the changes they might cause in the domain structures. The tool searches UNIProtKB database for motifs and in the produced result showed 9 Ankyrin Repeat domains in RNase L consisting of residues 24 to 329. RNase L also contains a protein kinase domain and the functional KEN domain from residues 365 to 586 and 589 to 723, respectively.

Mutations G59S and P62S are located in the ANK 2 repeat, L184 is located in ANK 5 repeat, L224P in ANK 6, and A276V in ANK 8 domain; whereas L361P is buried in a B-sheet and the remaining three mutations R592H, T595M, and I673T are located in the KEN domain. None of the selected nsSNPs in this study were in the protein kinase domain of RNase L.

**Figure 2:**
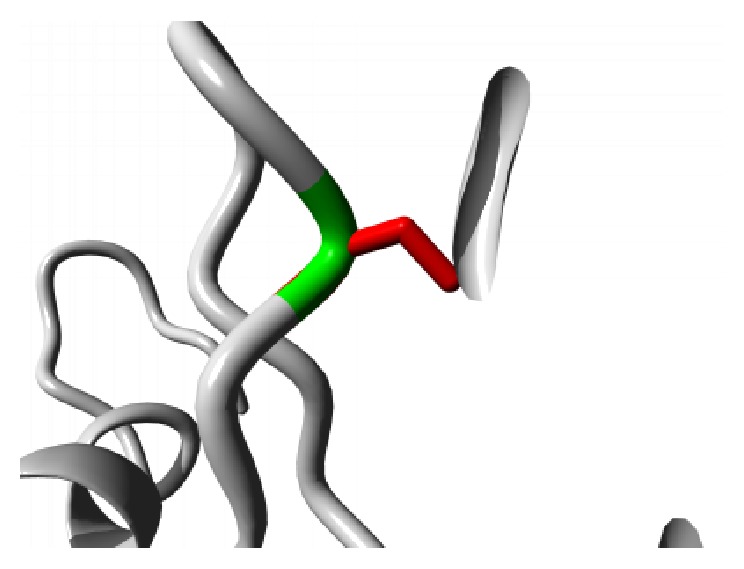
Close-up of the mutation G59S. The protein is colored grey; the side chains of both the wild type (Glycine) and the mutant (Serine) residue are shown and colored green and red, respectively.

The effects of the amino acid substitutions on the domain structure of the protein were received in detail from Project HOPE server. The mutation G59S results in a serine residue in place of Glycine at 59th position located in the ANK2 repeat region. This region is necessary for protein binding and alteration of the buried Glycine residue with larger Serine residue might abolish the core structure of the domain and affect protein binding. A graphical view of the mutation is shown in Figure 2.

**Figure 3:**
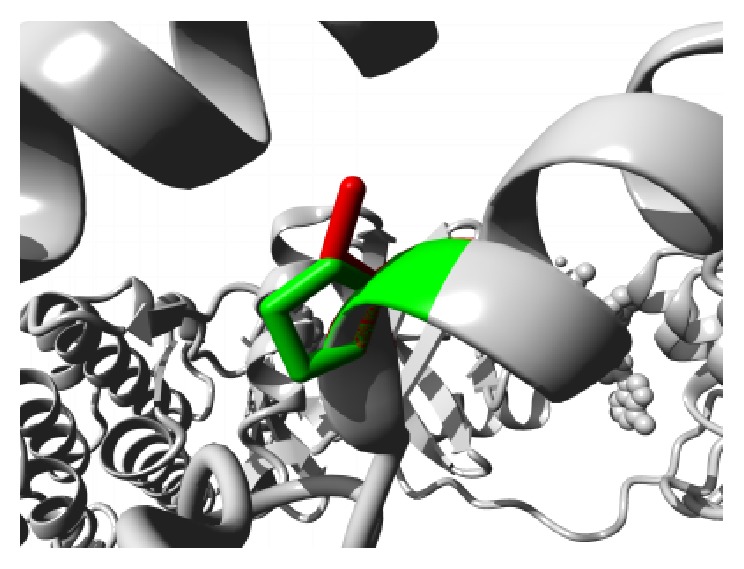
Close-up of the mutation P62S. The protein is colored grey; the side chains of both the wild type (Proline) and the mutant (Serine) residue are shown and colored green and red, respectively.

**Figure 4:**
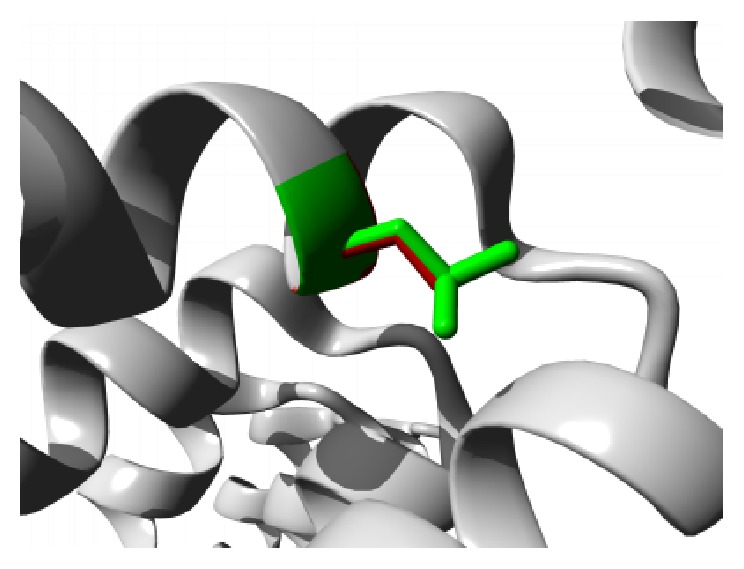
Close-up of the mutation L184S. The protein is colored grey; the side chains of both the wild type (Leucine) and the mutant (Serine) residue are shown and colored green and red, respectively.

**Figure 5:**
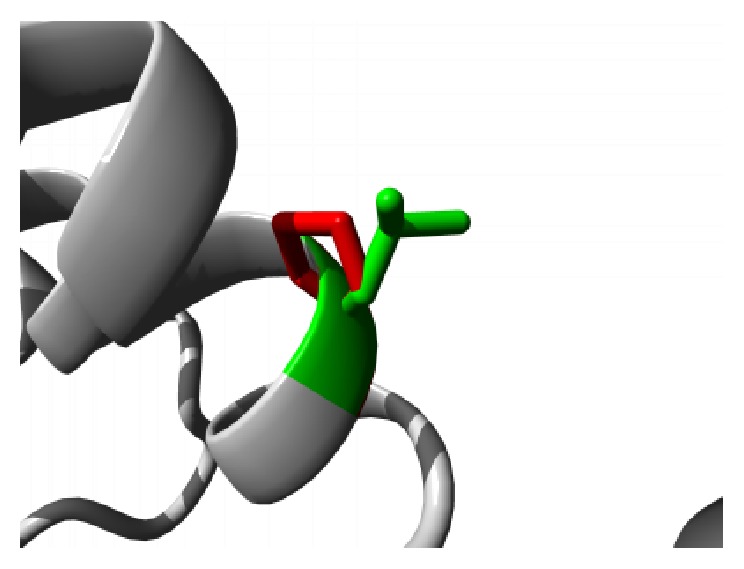
Close-up of the mutation L224P. The protein is colored grey; the side chains of both the wild type (Leucine) and the mutant (Proline) residue are shown and colored green and red, respectively.

**Figure 6:**
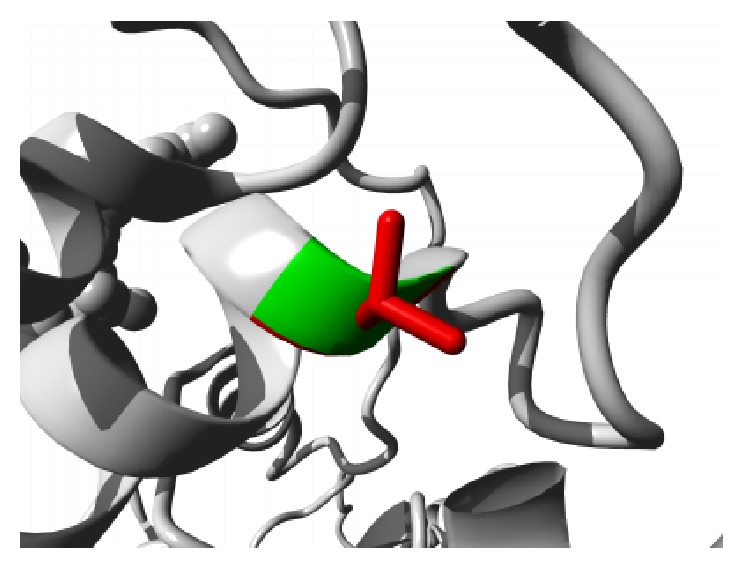
Close-up of the mutation A276V. The protein is colored grey; the side chains of both the wild type (Alanine) and the mutant (Valine) residue are shown and colored green and red, respectively.

Similar destabilizing condition is created by the mutation P62S. Replacing a buried Proline with a smaller Serine residue creates a hollow space in the core of the domain that might disturb the function of the domain as shown in Figure 3. In ANK6 repeat region mutation L184S also introduces a smaller residue than the wild type and the result alters the repeat region and hampers the function. The mutations are shown in Figure 4. The next mutation (L224P) disrupts an alpha helix in the protein located in ANK6 repeat. As the mutated residue is located on the surface of a binding domain and is smaller in size than the wild type it is likely to disturb the external interaction of the domain (Figure 5). Mutation A276V replaces Alanine residue on an ankyrin repeat domain (ANK8) to Valine. Valine is larger than Alanine which is buried and the size difference will probably hamper the core structure of the protein (Figure 6).

**Figure 7:**
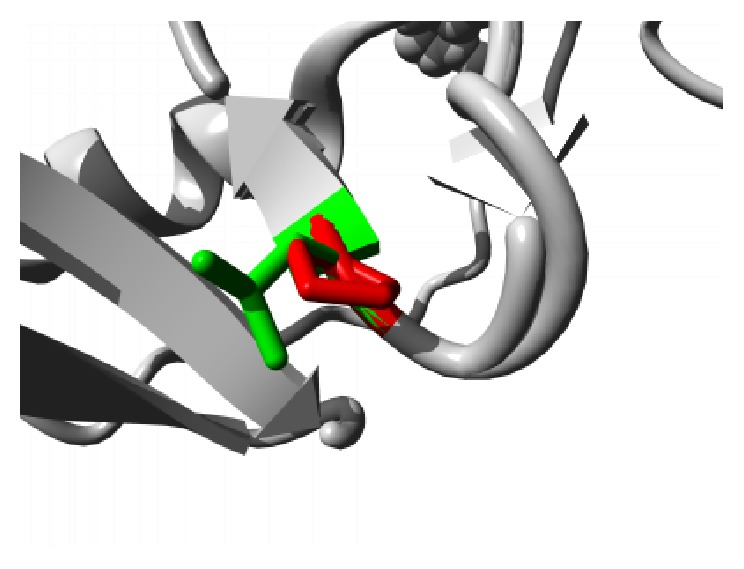
Close-up of the mutation L361P. The protein is colored grey, the side chains of both the wild type (Leucine) and the mutant (Proline) residue are shown and colored green and red, respectively.

**Figure 8:**
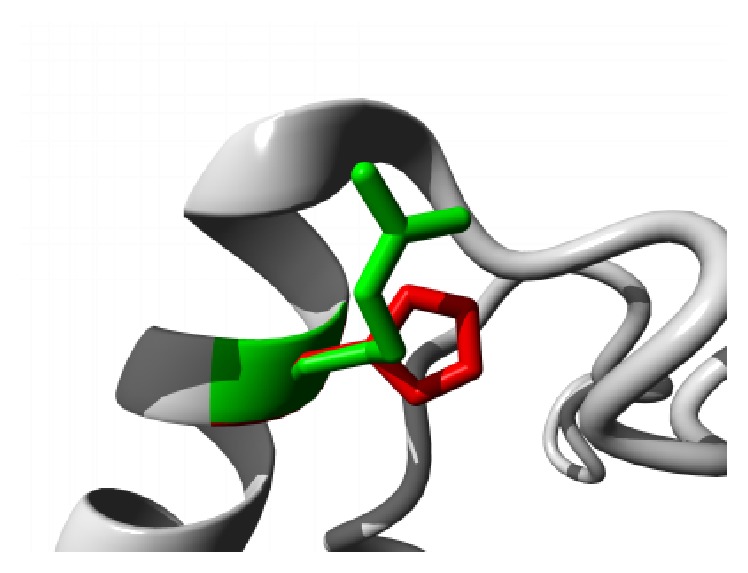
Close-up of the mutation R592H. The protein is colored grey; the side chains of both the wild type (Arginine) and the mutant (Histidine) residue are shown and colored green and red, respectively.

**Figure 9:**
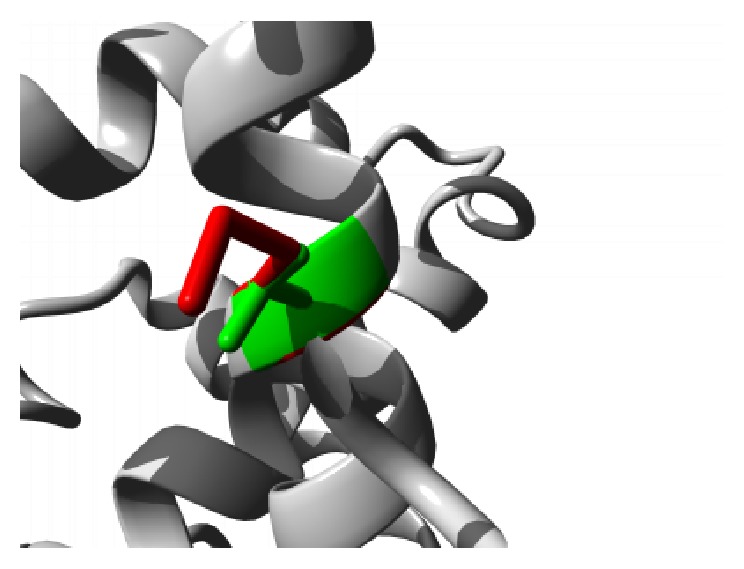
Close-up of the mutation T595M. The protein is colored grey; the side chains of both the wild type (Threonine) and the mutant (Methionine) residue are shown and colored green and red, respectively.

**Figure 10:**
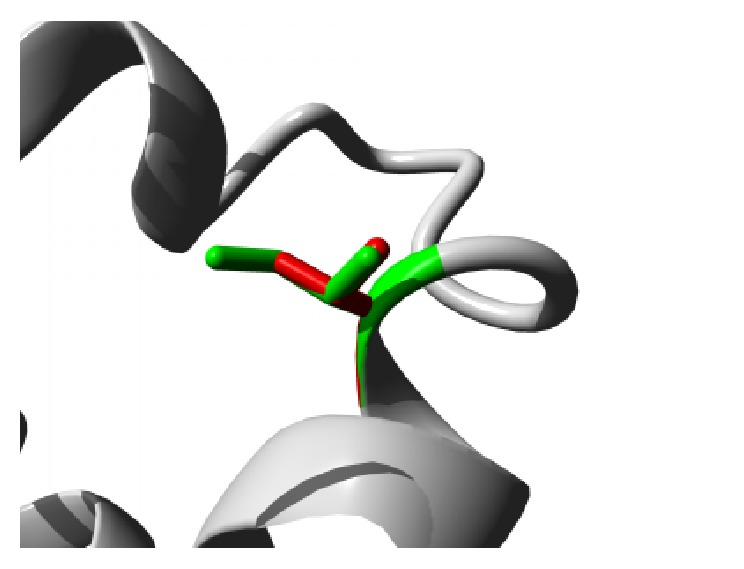
Close-up of the mutation I673T. The protein is colored grey; the side chains of both the wild type (Isoleucine) and the mutant (Threonine) residue are shown and colored green and red, respectively.

Protein loses flexibility when a flexible residue (Leucine) is replaced with a rigid one like Proline. Mutation L361P, as a result, can disrupt the core structure of the protein (Figure 7). The KEN domain of the protein RNase L is accounted for its endonuclease activity and a functional domain of the protein. The mutation at position 592 (R592H) replaces the wild type residue with a smaller residue. Residue 592 is exposed, so the mutation is likely to cause loss of functional property for replacing an exposed residue. Moreover, the wild type residue at position 592 formed a salt bridge with Aspartic Acid at 556 and Glutamic Acid at 457 and 558. The mutation will disturb the ionic interaction of the wild type residue and affect the catalytic activity of the domain. The mutation is shown in Figure 8. The other two mutations T595M and I673T in the KEN domain also abolish the function of the domain. Replacing the 595th residue Threonine with Methionine might cause loss of hydrogen bonding and incorrect folding as Methionine is bigger and more hydrophobic than Threonine. Similarly mutation I673T can also disrupt the function of the KEN domain by introducing a less hydrophobic and smaller residue (Isoleucine to Threonine) resulting in an empty space and loss of hydrophobic interaction in protein core. The results are shown in Figures 9 and 10.

## 4. Discussion

SNP stands for Single Nucleotide Polymorphism. These single nucleic acid variations are the major cause of variations among people; besides SNPs are also accounted for majority of inherited diseases as more than half of known genetic disorders involve amino acid substitutions. To date nearly four million SNPs could be found on NCBI SNP database but many SNPs do not pose any significant change or any change at all on protein structure and function due to degeneracy of amino acids and natural selection, which will ultimately remove mutations in functionally important regions. As a result genetic studies to distinguish between functionally neutral and disease associated polymorphisms have become a major affair. Since SNPs are dispersed throughout the genome they are excellent genetic markers and an inseparable utility in disease research. Although most disease associated SNPs are found in the exons or coding regions, also known as nonsynonymous SNPs, there is also evidence of SNPs occurring in the intronic regions of gene and disrupting the regulatory region which in turn affects splicing process and gene expression.

With the increasing number of reported and recorded SNPs in several databases expensive population based surveys have become challenging as the sheer number of SNPs data makes it difficult to choose a target SNP for study which are most likely to contribute in disease development.* In silico *approach in this situation is a handy way to distinguish the deleterious SNPs using specialized algorithms that can differentiate between neutral and deleterious SNPs by analyzing the databases and incorporating functional and structural evidence about the ultimate effect of a polymorphism. By using phylogenetic analysis and structural analysis of mutated proteins using hypothetical models can provide a fair amount of information with recognizable precision.

RNASEL resides in one of the prostate cancer (PRCA) susceptibility loci HPC1, discovered in 1996. In linkage analysis it is showed that a number of loci contain PRCA susceptibility genes and that implies the heterogeneous nature of the disease incorporating several genetic and environmental factors [79, 80].

Recent studies have found several accounts indicating RNASEL linkage to prostate cancer but there remains a significant amount of polymorphism data on RNASEL that awaits extensive population based and clinical studies. In this current study the SNP databases were analyzed to find out SNPs that might potentially be deleterious for RNASEL employing computational methods.

Search for nsSNPs against RNASEL resulted in 122 hits. The rsIDs were submitted to SIFT and PolyPhen-2 servers. SIFT found 11 nsSNPs as nontolerable and PolyPhen found 28 nsSNPs to be probably damaging (Table 1). 9 SNPs were predicted deleterious in common. Furthermore, we analyzed the data with several other SNP analyzing algorithms and in a combined result 9 nsSNPs were predicted deleterious or disease related by at least 6 or more out of the used 7 algorithms. The rsIDs and their corresponding mutations are listed in Table 2, among them rs151296858 (G59S) and rs35896902 (R592H) are previously known to be associated with the risk of prostate cancer [21, 81].

UTRScan results confirmed the experimental finding that there are SNPs in the UTR region of RNASEL associated with cancer predisposition. One 5′ UTR RNASEL SNP rs3738579 reportedly increases the risk of several cancers including prostate cancer. Three 3′ UTR SNPs, rs12135247, rs1048260, and rs11072, have also been reported in RNASEL. UTRScan provided a preview of the UTR region of RNASEL. From the results two SNPs (rs566253706, rs566253706) were found in the Mushashi Binding Elements (MBE) of 3′ UTR although no experimental evidence is available [82].

The protein data bank contained two structures (4OAV, 4OAU) with 100% identity with RNase L. We performed energy minimization and RMSD calculation of the mutant and wild type models. The free energy of all the mutated models decreased significantly from the wild type models. The RMSD also indicated significant change in mutant models. Highest RMSD 2.59 was score by rs35896902 (R592H) and lowest calculated for rs142939718 (L361P) was 1.11. Other RMSD values ranged from 1.14 to 1.94. According to RMSD value the mutation R592H causes the most deviation from the wild type structure of RNase L followed by A276V (1.94).

The lowest total free energy after energy minimization was −431021.9 kj/mol for mutation A276V followed by −422287.5 kj/mol for T595M. Significant decrease in free energy is recorded for all the mutation. Considering the RMSD value and decrease being free energy mutation A276V seems to cause notable deviation from the wild type model of RNase L. The other mutation R592H which is also found to be associated with PRCA in experimental data was also found to be more deleterious than other mutations in our study. This finding is also supported by the phylogenetic analysis data from ConSurf. The 276th residue Alanine is a buried structural residue, with conservation score 9, denoting it is a crucial change in protein stability due to the mutation A276V according to Project HOPE.

Residue Alanine 592 is an exposed functional residue in the KEN domain of the protein and probably the mutation R592H will result in loss of catalytic activity of the domain as predicted by Project HOPE.

Although, all the mutations are likely to disrupt protein function and structure; mutations G59S, P62S, and L361P along with previously mentioned L224P and R592H are most likely do cause severe dysfunction as all the residues are strictly conserved and either functionally or structurally involved.

The RNase L synthesis is induced by interferon upon viral infection. After binding with 2-5A (5′-phosphorylated 2′,5′-linked oligoadenylates) activation occurs through subsequent homodimerization. Now, the ankyrin repeat domains are also called 2-5A sensors and they constitute the 2-5A binding domain. As a result, the mutations in the ankyrin repeat regions (G59S, P62S, L184S, L224P, A276V, and L361P) can hamper RNase L activation by hampering 2-5A binding. The formation and stability of the homodimer depend on ankyrin repeat domains and kinase domain of the protein. Although no mutations in the kinase domain remain in the final list of damaging SNPs in this study, all the mutations in ANK region potentially causes instability in protein structure as discussed above and shown by Project HOPE analysis. The other mutations (R592H, T595M, and I673T) in the ribonuclease domain of the protein can possibly inhibit the catalytic function of the protein. Mutation R592H replaces a conserved and exposed residue in the functional domain. The molecular analysis of this amino acid substitution showed this mutation can substantially destabilize the functional capability of RNase L because this substitution eliminates several molecular interactions of the wild type Arginine residue. According to the molecular analysis the mutations might either retard structural integrity and produce or interfere with the functional features which ultimately lead to inhibition of RNase L activity.

So far, studies involving RNASEL nsSNPs have been done in several populations but only with a few numbers of SNPs under consideration. A study on German population found mutations E265X and R400P to be involved in PRCA susceptibility [83].

E265X is also reported in several studies in Finnish, German, and Swedish populations [81, 83, 84]. The substitution R462Q is a common variant reported to both increase [73] and decrease the susceptibility to PRCA [85]. D541E, a missense variant, was found associated with increased PRCA risk in Japan [85] and several other studies found no association [81, 86, 87]. But none of these mutations except G59S and R592H [21, 81] met the selection criteria of this current* in silico* analysis because several SNP analyzing algorithms failed to predict their deleterious effects and nonsense variants (E265X) were not taken into consideration in this study. Nevertheless, the deleterious nsSNPs that are found in this study, especially rs151296858 (G59S), rs145415894 (A276V), 11 and rs35896902 (R592H); two out of these three mutations have been reported in population based studies and proved to be potentially destabilizing both functionally and structurally in our analysis. In the process of this study it was seen that despite some correct assumption the web-based tools need to be more precise in detecting deleterious SNPs and population based studies are necessary to identify and test the predicted SNPs in different populations.

## 5. Conclusion

In this study available data from the NCBI dbSNP database for the prostate cancer susceptibility gene RNASEL has been analyzed through several SNP analyzing tools and the predicted deleterious SNPs were evaluated for their potential deleterious effect on the protein function and stability. Nine SNPs were predicted deleterious; those are rs151296858, rs114166108, rs182539049, rs138685180, rs145415894, rs142939718, rs35896902, rs147890567, and rs150721457; among them three nsSNPs rs151296858 (G59S), rs145415894 (A276V), and rs35896902 (R592H) have the most probability to increase PRCA susceptibility. The predicted SNPs are located in the ANK repeat domains of the protein which facilitate binding of other molecules and the catalytically active KEN domain which bears the ribonuclease activity of the protein. So, it is very likely that there are unreported nsSNPs increasing disease predisposition by altering protein function or structure. The findings of this study will hopefully help to distinguish the damaging SNPs which increase the risk of prostate cancer in patients of different populations' in future genome-wide studies. Therefore, extensive population based studies and clinical studies are required to characterize the vast SNP data and for the verification of the findings of the current study.

## Figures and Tables

**Table 1:** List of SNPs predicted by SIFT as damaging.

SNP	Position	Amino acid change	Prediction	Score	Median
rs145581875	50	N50S	Damaging	0.02	2.81
rs114166108	62	P62S	Damaging	0.02	2.81
rs182539049	184	L184S	Damaging	0.01	2.81
rs146781980	195	V195I	Damaging	0.02	2.81
rs145415894	276	A276V	Damaging	0.01	2.81
rs142939718	361	L361P	Damaging	0.02	2.78
rs145787003	406	S406F	Damaging	0.01	2.81
rs190359946	483	H483Q	Damaging	0.02	2.91
rs35896902	592	R592H	Damaging	0.01	3.1
rs143374873	727	C727Y	Damaging (low confidence)	0.03	3.63
rs150721457	673	I673T	Damaging	0.05	3.12

**Table 2:** List of SNPs that were predicted by PolyPhen-2 server as significantly damaging.

rsIDs	Amino acid position	Mutation	PSIC score	Effect
rs192719507	44	L44F	1	Probably damaging
rs151296858	59	G59S	1	Probably damaging
**rs114166108**	**62**	**P62S**	**0.997**	**Probably damaging**
rs113469614	65	N65K	0.989	Probably damaging
rs141809307	94	T94T	1	Probably damaging
rs56250729	97	I97L	0.997	Probably damaging
rs138740835	98	L98F	0.539	Possibly damaging
rs115634589	101	I101T	0.999	Probably damaging
rs182539049	184	L184S	0.992	Probably damaging
**rs146781980**	**195**	**V195I**	**0.98**	**Probably damaging**
rs138685180	224	L224P	0.999	Probably damaging
rs143565946	258	L258H	0.999	Probably damaging
**rs145415894**	**276**	**A276V**	**1**	**Probably damaging**
rs140715742	285	L285M	0.995	Probably damaging
rs35553278	289	A289T	0.591	Possibly damaging
rs151056294	318	K318T	0.651	Probably damaging
rs148464936	355	R355C	1	Probably damaging
**rs142939718**	**361**	**L361P**	**1**	**Probably damaging**
**rs145787003**	**406**	**S406F**	**0.976**	**Probably damaging**
rs486907	462	R462Q	0.998	Probably damaging
**rs190359946**	**483**	**H483Q**	**1**	**Probably damaging**
rs148411578	528	V528I	0.982	Probably damaging
rs193195484	532	V532I	0.999	Probably damaging
**rs35896902**	**592**	**R592H**	**1**	**Probably damaging**
rs147890567	595	T595M	1	Probably damaging
rs142133260	601	N601S	1	Probably damaging
**rs150721457**	**673**	**I673T**	**1**	**Probably damaging**

PSIC = position-specific independent count score.

[Damaging rsID predicted by both SIFT and PolyPhen-2 server are shown in bold letters].

**Table 3:** List of SNPs predicted to be disease related by 7 SNP analyzer algorithms. (T = tolerated, as predicted by SIFT.)

rsIDs	Mutation	PolyPhen-2 score	SIFT score	nsSNPs	PhD-SNP	PANTHER	SNPs&GO	P-Mut
rs151296858	G59S	1	T	Disease	Disease	Disease	Disease	—
rs114166108	P62S	0.997	0.02	Disease	Disease	Disease	Neutral	—
rs182539049	L184S	0.992	0.01	Disease	Disease	Disease	Neutral	—
rs138685180	L224P	0.999	T	Disease	Disease	Disease	Disease	Pathological
**rs145415894**	A276V	1	0.01	Disease	Disease	—	Neutral	Pathological
rs142939718	L361P	1	0.02	Disease	Disease	Disease	Disease	Pathological
**rs35896902**	R592H	1	0.01	Disease	Disease	Disease	Disease	Pathological
rs147890567	T595M	1	T	Disease	Disease	Disease	Neutral	Pathological
rs150721457	I673T	1	0.05	Disease	—	—	Disease	—

**Table 4:** Conservation profile of amino acids in RNASEL that coincide in location with high-risk nsSNPs.

rsIDs	Residue pos.	Residue	CS	Color	Buried or exposed	Function
rs151296858	59	G	−0.834	8	e	f
rs114166108	62	P	−0.99	9	e	f
rs182539049	184	L	−0.296	6	b	
rs138685180	224	L	−0.146	6	b	
**rs145415894**	276	A	−1.202	9	b	s
rs142939718	361	L	−1.16	9	b	s
**rs35896902**	592	R	−0.915	8	e	f
rs147890567	595	T	−0.345	6	b	
rs150721457	673	I	−0.552	7	b	

CS: conservation score (1–4 = variable, 5 = average, and 6–9 = conserved); (f): predicted functional site; (s): predicted structural site.

**Table 5:** Result showing UTR regions in RNASEL transcript from UTRScan server.

Signal name	UTR region	Match total	Position in transcript
BRD_BOX	3′	1	2679–2685

GY_BOX	3′	1	39–45

uORF (upstream open reading frames)	5′	13	—

MBE (Musashi Binding Element)	3′	4	901–905
			3132–3136
			3231–3455
			4220–4258

PAS (Polyadenylation Signal)	3′	1	4220–4258

**Table 6:** RMSD (Å) values and total free energy after energy minimization of the wild type and mutated protein models. RMSD calculated by Tm-Align and energy minimization was performed by YASARA force field.

Mutated models	Energy after minimization (kj/mol)	RMSD (Å)
Wild Type	−436437.8	
R592H	−393543.3	2.59
P361	−396456.0	1.11
G59S	−399615.0	1.19
P62S	−405223.0	1.14
A276V	−431021.9	1.94
L184S	−403094.0	1.74
L224P	−402604.0	1.57
T595M	−422287.5	1.45
I673T	−403967.2	1.48

**Table 7:** I-mutant predictions for selected nsSNPs.

Mutation	Sign of DDG	DDG value prediction kcal/mol	RI	RSA
G59S	Decrease	−1.36	5	3.8
P62S	Decrease	−2.15	8	0.7
L184S	Decrease	−2.24	5	0.0
L224P	Decrease	−2.13	9	15.8
A276V	Increase	0.66	3	0.9
L361P	Decrease	−1.59	8	0.0
R592H	Decrease	−0.99	9	6.6
T595M	Decrease	−0.54	7	2.2
I673T	Decrease	−2.46	9	9.2

DDG: free energy change value in Kcal/mol (>0 increase, <0 decrease, >0.5 large increase, and <2.5 large decrease); sign of DDG: the direction of the change (increase or decrease); the reliability index (RI) from 0 to 9 is shown in parentheses, where 0 is the lowest RI and 9 is the highest; RSA: relative surface accessibility.
